# Supplementary material for: Assessment the Impacts of Sea-Level Changes on Mangroves of Ceará-Mirim Estuary, Northeastern Brazil, during the Holocene and Anthropocene
Source: Plants (Basel). 2023 Apr 20;12(8):1721. doi: 10.3390/plants12081721 (PMC10141466; doi:10.3390/plants12081721)
Supplement: Supplementary file 1 [file plants-12-01721-s001.zip › plants-2161456-supplementary.pdf]

Table S1. Age-depth model using Bayesian statistics to reconstruct accumulation history for sedimentary deposit of core NAT-5.

Depth	Min Age	Max Age	Median Age	Mean Age	Depth	Min Age	Max Age	Median Age	Mean Age
**0**	-80	-52	-67	-67	**76**	2782	3056	2893	2897
**1**	-68	47	-36	-29	**77**	2795	3095	2920	2924
**2**	-64	157	-6	9	**78**	2804	3155	2945	2951
**3**	-60	267	24	46	**79**	2811	3217	2968	2979
**4**	-57	377	54	84	**80**	2817	3286	2991	3006
**5**	-54	487	84	121	**81**	2841	3315	3019	3033
**6**	-29	530	124	160	**82**	2856	3338	3048	3060
**7**	-13	573	165	198	**83**	2868	3375	3076	3088
**8**	-1	627	206	236	**84**	2879	3421	3103	3115
**9**	9	706	244	274	**85**	2891	3482	3127	3142
**10**	19	786	279	312	**86**	2919	3506	3156	3169
**11**	53	823	320	352	**87**	2941	3528	3184	3197
**12**	77	862	362	392	**88**	2956	3562	3212	3224
**13**	93	918	405	431	**89**	2972	3597	3240	3252
**14**	108	974	444	471	**90**	2985	3646	3267	3279
**15**	119	1055	482	511	**91**	3016	3667	3294	3306
**16**	157	1089	521	549	**92**	3038	3691	3323	3334
**17**	183	1133	559	588	**93**	3055	3719	3350	3361
**18**	208	1179	602	627	**94**	3070	3754	3378	3388
**19**	226	1241	637	665	**95**	3082	3800	3407	3416
**20**	242	1304	676	704	**96**	3114	3820	3434	3442
**21**	284	1334	717	742	**97**	3138	3845	3463	3469
**22**	320	1377	758	780	**98**	3160	3871	3492	3496
**23**	348	1408	799	818	**99**	3181	3901	3519	3522
**24**	372	1451	837	856	**100**	3197	3940	3546	3549
**25**	390	1500	873	895	**101**	3229	3956	3574	3576
**26**	438	1525	914	934	**102**	3263	3976	3601	3604
**27**	479	1564	956	974	**103**	3286	3996	3631	3631
**28**	504	1607	1000	1014	**104**	3307	4023	3659	3658
**29**	531	1653	1042	1054	**105**	3323	4055	3687	3686
**30**	548	1715	1082	1093	**106**	3355	4069	3714	3713
**31**	586	1743	1122	1133	**107**	3380	4088	3742	3740
**32**	622	1775	1165	1173	**108**	3405	4113	3771	3768
**33**	655	1809	1206	1212	**109**	3423	4139	3800	3795
**34**	681	1846	1248	1252	**110**	3442	4172	3828	3822
**35**	706	1900	1287	1291	**111**	3480	4188	3856	3850
**36**	750	1931	1327	1330	**112**	3514	4208	3885	3877
**37**	787	1957	1370	1369	**113**	3545	4229	3914	3905
**38**	820	1991	1409	1408	**114**	3569	4256	3941	3932

**39**	849	2028	1450	1448	**115**	3588	4282	3968	3960
**40**	876	2076	1489	1487	**116**	3632	4295	3994	3987
**41**	929	2105	1529	1526	**117**	3671	4310	4024	4015
**42**	966	2136	1569	1565	**118**	3702	4330	4051	4043
**43**	999	2169	1611	1604	**119**	3724	4360	4080	4070
**44**	1039	2200	1654	1643	**120**	3743	4388	4108	4098
**45**	1066	2238	1691	1682	**121**	3792	4400	4134	4126
**46**	1121	2260	1734	1722	**122**	3839	4416	4161	4153
**47**	1168	2289	1773	1762	**123**	3878	4430	4188	4181
**48**	1219	2318	1814	1802	**124**	3901	4450	4219	4209
**49**	1253	2366	1855	1843	**125**	3922	4477	4247	4236
**50**	1281	2414	1896	1883	**126**	3984	4484	4271	4263
**51**	1338	2434	1933	1921	**127**	4044	4492	4294	4290
**52**	1387	2461	1973	1959	**128**	4087	4503	4320	4317
**53**	1419	2490	2013	1998	**129**	4128	4518	4348	4343
**54**	1456	2525	2053	2036	**130**	4157	4540	4376	4370
**55**	1488	2567	2091	2074	**131**	4173	4565	4398	4391
**56**	1549	2586	2132	2114	**132**	4189	4603	4418	4413
**57**	1614	2605	2173	2153	**133**	4202	4645	4437	4434
**58**	1659	2631	2212	2193	**134**	4215	4697	4455	4455
**59**	1693	2665	2252	2232	**135**	4226	4759	4473	4476
**60**	1725	2707	2295	2272	**136**	4247	4777	4494	4496
**61**	1790	2718	2331	2311	**137**	4262	4805	4512	4516
**62**	1852	2734	2371	2351	**138**	4273	4833	4531	4536
**63**	1904	2756	2411	2390	**139**	4289	4870	4549	4556
**64**	1931	2786	2455	2430	**140**	4298	4905	4567	4576
**65**	1959	2820	2494	2469	**141**	4318	4931	4588	4597
**66**	2045	2835	2534	2510	**142**	4329	4958	4606	4617
**67**	2132	2853	2574	2551	**143**	4340	4984	4627	4637
**68**	2198	2871	2615	2592	**144**	4350	5016	4648	4657
**69**	2239	2897	2658	2633	**145**	4362	5050	4667	4677
**70**	2276	2926	2703	2674	**146**	4382	5067	4687	4698
**71**	2385	2939	2732	2713	**147**	4402	5088	4708	4718
**72**	2489	2955	2762	2752	**148**	4415	5114	4730	4738
**73**	2596	2974	2793	2791	**149**	4426	5151	4749	4758
**74**	2693	3000	2828	2830	**150**	4441	5187	4767	4779
**75**	2759	3028	2865	2870					
